# Application of Clinical Decision Support System to Assist Breast Cancer Patients with Lifestyle Modifications during the COVID-19 Pandemic: A Randomised Controlled Trial

**DOI:** 10.3390/nu13062115

**Published:** 2021-06-20

**Authors:** Panos Papandreou, Aristea Gioxari, Frantzeska Nimee, Maria Skouroliakou

**Affiliations:** 1 Department of Nutrition, IASO Hospital, 15123 Athens, Greece; ppapandreou@cibusmed.com; 2 Department of Dietetics and Nutritional Science, School of Health Science and Education, Harokopio University, 17671 Athens, Greece; fnimee@hua.gr (F.N.); mskour@hua.gr (M.S.)

**Keywords:** clinical decision support systems, breast cancer, Mediterranean diet, obesity, quality of life, cardiovascular risk factors, oxidative stress

## Abstract

Clinical decision support systems (CDSS) are data aggregation tools based on computer technology that assist clinicians to promote healthy weight management and prevention of cardiovascular diseases. We carried out a randomised controlled 3-month trial to implement lifestyle modifications in breast cancer (BC) patients by means of CDSS during the COVID-19 pandemic. In total, 55 BC women at stages I-IIIA were enrolled. They were randomly assigned either to Control group, receiving general lifestyle advice (*n* = 28) or the CDSS group (*n* = 27), to whom the CDSS provided personalised dietary plans based on the Mediterranean diet (MD) together with physical activity guidelines. Food data, anthropometry, blood markers and quality of life were evaluated. At 3 months, higher adherence to MD was recorded in the CDSS group, accompanied by lower body weight (kg) and body fat mass percentage compared to control (*p* < 0.001). In the CDSS arm, global health/quality of life was significantly improved at the trial endpoint (*p* < 0.05). Fasting blood glucose and lipid levels (i.e., cholesterol, LDL, triacylglycerols) of the CDSS arm remained unchanged (*p* > 0.05) but were elevated in the control arm at 3 months (*p* < 0.05). In conclusion, CDSS could be a promising tool to assist BC patients with lifestyle modifications during the COVID-19 pandemic.

## 1. Introduction

The most commonly diagnosed cancer worldwide is now breast cancer (BC), with over 2 million new cases (11.7%) reported during 2020 [1]. Lifestyle, as well as environmental, genetic and hormonal factors have been suggested to contribute to BC development [2]. Obesity is considered as a prime risk factor for postmenopausal BC and has been associated with low BC-specific survival [3] and all-cause mortality [4]. Furthermore, the risk of BC recurrence rises in the presence of obesity [5] with negative effects on treatment efficiency [6]. One underlying mechanism that could explain the link between obesity and BC is the disrupted redox homeostasis, which is commonly present in breast and adipose tissues of BC women [7]. Weight gain after diagnosis is also usual in BC and has been associated with greater all-cause mortality rates when compared to BC women of unaltered body weight [8]. Several physiological and behavioral factors, such as chemotherapy treatment, menopause, alterations in body composition, metabolic changes, inactivity, as well as fatigue and depression have been suggested to lead to post-diagnosis weight gain [9]. What is more, BC women are at high risk of developing comorbidities related to obesity, including cardiovascular diseases (CVD), which may further negatively affect disease prognosis [10]. 

Lifestyle has a significant impact on both BC prevention and progression [11,12]. For instance, physical activity engagement following BC diagnosis such as walking, biking, swimming, has been shown to reduce the risk of cancer recurrence [13,14]. Furthermore, diet patterns may influence mental and physical functioning affecting overall health-related quality of life [15]. Nutritional education for weight management and support for dietary change are beneficial for BC patients [16]. In a recent randomised controlled trial of our research team, BC patients following the Mediterranean Diet (MD) showed significant improvements towards body mass index (BMI), body fat mass (BFM), blood concentration of antioxidant vitamin C, as well fasting blood glucose levels [17]. The Mediterranean dietary pattern is characterised by an abundance of plant foods, fish and especially olive oil [18]. 

Treatment of primary cancer involves initial surgery followed by radiation therapy, chemotherapy, antiestrogen therapy or targeted therapy [19]. The application of Clinical Decision Support Systems (CDSS) is a major progress in medical practice assisting physicians in the process of making clinical decisions for BC treatment [20]. CDSS are data aggregation tools based on software technologies that are designed to aid healthcare staff in a broad range of clinical practices, such as prescribing treatments, clinical guidelines and patient screening [21]. CDSS are used to ameliorate decision timing and patient health-related quality of life reducing both costs and error rates in medical care [22,23]. Currently, CDSS have been designed to promote compliance to lifestyle guidelines for healthy weight management and prevention of cardiovascular diseases [24,25,26]. Patient assessment and treatment recommendations are personalised according to individuals’ incorporated data, which are entered through an electronic health record (EHR) system [24].

Considering the impact of Covid-19 pandemic on cancer care, the Greek Ministry of Health and the Greek National Health Service Organization (EOPYY) proceeded to adjustments in healthcare units to facilitate access to cancer care and limit the exposure of cancer patients to the risk of infection. Within this scope, our research team developed a food database CDSS enabling BC patients to follow lifestyle guidelines at the point of care at home. In the present randomised controlled 3-month trial, we investigated the effectiveness of the food database CDSS in the degree of MD adherence, quality of life and nutritional status of BC patients, during the first wave of COVID-19 pandemic in Greece (Spring to Fall 2020).

## 2. Materials and Methods

### 2.1. Ethics

The study was reviewed and approved by the Ethics Committee of IASO HOSPITAL in Athens-Greece (Approval Code #D31052019). The trial was conducted according to the principles of Helsinki Declaration (1964) and was in line with terms of Good Clinical Practice. ClinicalTrials.gov registry: NCT04876560.

### 2.2. Participants

Adult BC outpatients of IASO HOSPITAL in Athens-Greece were initially invited to take part in the study through an announcement at the hospital’s web site. Patients, who showed interest in participating, attended personal meetings with the appointed dieticians who explained in detail the aims, methods and the potential benefits/risks of the study. A leaflet with related information was provided to all patients. Before recruitment, each eligible volunteer signed a written informed consent and then kept a hard copy of the signed document. All eligible patients were recruited from March to June 2020.

Inclusion criteria: ≥ 18 years of age women with histological evidence of primary invasive breast cancer at stages I-IIIA, who underwent mastectomy followed by antiestrogen therapy, were enrolled in the study. Additional criterion was a good performance status, as indicated by scoring "0 or 1" of the Eastern Cooperative Oncology Performance Status (ECOG PS) questionnaire [27]. Exclusion criteria: history of any other type of cancer during the last 5 years; suffering from severe illness such as organ failure (e.g., heart, liver, renal), autoimmune diseases (e.g., Crohn’s disease) or congenital metabolic disorders; emerging health issues (e.g., undergone surgery, malabsorption, infection) that could impede the conduct of the trial; diagnosis of severe psychiatric disease; alcoholism; addiction to drugs; taking medications to treat obesity; following a plant based diet during the last 5 years; the use of nutrient or non-nutrient supplements for the last 6 months.

### 2.3. Study Design

A team of scientists (pharmacists, dieticians, physicians, liberal art and marketing communications consultants) at IASO HOSPITAL (Athens, Greece) developed a food database CDSS, that could provide the following services: (I) nutritional status assessment (screening); (II) individual dietary plans according to patients’ needs; (III) nutritional goals and guidelines, coupled with appropriate definitions, explanations and healthy dietary practices; (IV) physical activity goals; (V) monitoring nutritional and physical activity goals. In the present study, the CDSS intended to assist BC patients with lifestyle modifications through the implementation of the Mediterranean diet (MD) and increase of physical activity. The CDSS was developed in 2016 and ever since, it has been applied in clinical practice to assist dieticians in patient nutritional screening. However, this is the first time that individuals themselves got access to CDSS from home, as a means of guidance to comply with lifestyle changes during the COVID-19 pandemic.

The present study was a parallel group, single centered, 3-month intervention trial in which a computer-generated simple randomisation sequence was implemented. The leading investigator recorded the code number along with the type of treatment for each participant and sealed data into an envelope. Allocation to treatment was blinded to the data scientist until the assessment of study results. 

Enrolled BC patients were assigned to either the Control or the CDSS (intervention) group. At baseline before the start of the trial (time 0), each enrolled participant completed a personal interview with the appointed dieticians. In the Intervention group (CDSS group), patients received a personalised daily dietary plan (specific meals, products, recipes, food portions in grams) based on MD together with physical activity guidelines, all generated by the CDSS. Body mass index (BMI) was calculated by the CDSS, as the ratio of the average recorded weight (kg) to the square of average height (m^2^) [28]. The CDSS estimated Total daily Energy Expenditure (TEE) (Figure S1) according to individual’s basic metabolic rate (Harris–Benedict equation) and physical activity levels [29,30]. To this point, there was a “Lifestyle” section in the CDSS (Figure S2), in which physical activity status was distinguished in five categories, i.e., “Limitation to activities due to disability”, “Low active”, “Moderately active”, “Active” and “Vigorous active”. A detailed description, including hours of sitting and exercising and even kind of exercises, was recorded for each category. The concept of metabolic equivalent (MET) was used to evaluate physical activity intensity [30] The CDSS also assessed nutritional status using the “Malnutrition Universal Screening Tool” (MUST) (Figure S3), in order to identify adults who were at risk of malnutrition (undernutrition), malnourished or obese [31]. Overweight and obese BC patients received a hypocaloric diet, in which daily energy intake was less than TEE by approximately -500 kcal/day. Daily nutrient distribution was calculated by the CDSS taking into consideration the traditional MD pattern along with patients’ needs. More specifically, total protein intake ranged between 1.0 and 1.5 g per kg of body weight (BW). Total fat intake accounted for about 30% of total energy input and comprised less than 10% saturated fatty acids (SFAs), about 10% monounsaturated fatty acids (MUFAs) and about 10% polyunsaturated fatty acids (PUFAs). The daily amount of fiber consumption accounted for 20–30 g. Other health issues such as constipation, diarrhoea or gastro-esophageal reflux were also assessed by the CDSS for the formulation of the dietetic scheme. Furthermore, the dieticians provided nutritional consultation on MD [32]. Patients were trained to use the CDSS and received individual login passwords allowing access to their personal profile from home. During the trial, BC women were instructed to record food diaries in the CDSS every week (at least 2 weekdays and 1 weekend day), which were also made available to the dieticians (remotely). Visiting the CDSS, patients had the opportunity to track their progress, e.g., monitoring goals of body weight, physical activity, consumption of fruits, vegetables, legumes. Regular phone interviews were scheduled every 15 days to assist nutritional and lifestyle consultation, while unexpected phone calls were made to receive 24-hour dietary records.

The Control arm received general lifestyle advice according to “American Cancer Society Guidelines on Nutrition and Physical Activity for Cancer Prevention” [33] via scheduled phone interviews every 15 days. Food diaries of each week (at least 2 weekdays and 1 weekend day) were sent via emails and unexpected phone calls were made to receive 24-h dietary records as well. 

Throughout the intervention, each patient (of both groups) conducted two personal sessions with the appointed investigators, one at the trial initiation and one at 3 months (end of the study).

### 2.4. Assessments 

Medical record: General information (date of birth, smoking habits, allergies, gastrointestinal problems, other health issues), as well as detailed data on type and stage of breast cancer, performed surgery, hormone treatment and symptomatology were recorded for each patient by the appointed oncologist. 

Food data: To evaluate dietary patterns a Food Frequency Questionnaire (FFQ) was applied to all participants [34]. The FFQ assessed frequency of consumption (within a month) and portion sizes of foods and drinks typically consumed in MD. Pictures and food models were demonstrated to facilitate estimation of portion sizes. Nutrient intake was estimated by the collected food diaries and the 24-h dietary records using the Diet Analysis Plus software (version 6.1, Esha Research, Salem, MA, USA). The MedDietScore questionnaire estimated the degree of adherence to MD at the start of the study and at 3 months [35]. Scoring ranges from “0” to “55” and higher scores signify greater MD adherence.

Questionnaires: The Eastern Cooperative Oncology Group (ECOG) performance status (PS) was used to estimate patients’ general functional capacity and ability to perform daily activities [27]. The questionnaire comprises 5 point scores ranging from “0”, fully active, to “4”, completely disabled. A good PS corresponds to scoring “0” or “1" and indicates well-functioning [27]. Physical activity was assessed by the “International Physical Activity Questionnaire”. It evaluates frequency (number of days) and duration (minutes per day) of performed physical activities, including walking, vigorous and moderate intensity exercises, as well as sitting hours, during the last 7 days prior to screening [36]. Physical activity levels were expressed as METs-min per week. 

Health related quality of life was evaluated by two validated questionnaires: (a) the European Organization for Research and Treatment of Cancer Quality of Life Questionnaire Core 30 (EORTC QLQ-C30) and (b) the breast cancer supplementary module, EORTC QLQ-BR23 [37]. The EORTC QLQ-C30 (version 3.0, EORTC Data Center, Brussels, Belgium) comprises 30 items in blocks of three different scales: (i) global health status / quality of life (Qol), (ii) functional and (iii) symptom scales [37]. The EORTC QLQ-BR23 (EORTC Data Center, Brussels, Belgium) incorporates 23 items evaluating functional characteristics and symptomatology that are BC-specific, namely body image, sexual functioning, sexual enjoyment, future perspective, systemic therapy side effects, breast symptoms, arm symptoms and distress from hair loss [37]. Each item of EORTC QLQ-C30 and BR23 was transformed on a 0–100 scale. Greater scores for functional scales and global health status/Qol, and lower scores on symptomatology indicate overall good health and well-being. To this end, responses to financial difficulties, sexual enjoyment and feeling towards hair loss were not analysed in the present study, since over 50% of the enrolled participants refused to answer the corresponding items. Furthermore, we implemented the Hospital Anxiety and Depression Scale (HADS) to assess patients’ psychological distress [38]. It includes 14 self-assessed items of which seven questions are depression-specific and seven questions are anxiety-specific. Scoring ranges from “0”to “21” for each category and scores higher than “7” indicate possible cases of anxiety or depression, respectively [39]. All assessments (excluding ECOG PS) were performed at baseline (time 0) and at 3 months (follow up).

Anthropometric measurements: The BOD POD, an air displacement plethysmography device (BOD POD® Body Composition Tracking Systems, Life Measurement, Inc., Rome, Italy), was applied to measure body weight (BW) and body fat mass (BFM) as has been previously described [32]. Patients’ height was recorded to the nearest millimeter using a standard stadiometer. Waist circumference (WC) was measured to the nearest millimeter at the midpoint between the lower margin of the last palpable rib and the top of the iliac crest with a stretch-resistant measuring tape. We calculated body mass index (BMI) by dividing body mass (kg) to the square of height (m^2^), in order to define overweight and obese patients. All measurements were performed in the morning after overnight fasting both at study initiation and at 3 months. 

Blood indices: After overnight fasting, blood samples (~20 mL) were drawn from each patient at 0 and 3 months placing a catheter in an antecubital vein. Blood collection tubes containing ethylenediamine tetraacetic acid (EDTA) were used for plasma separation. Whole blood samples were allowed to clot at room temperature to isolate sera. Centrifugation was set at 3000 rpm for 10 min at 4 °C. Freshly isolated plasma or serum samples were used for all analyses. 

A biochemical analyser (Cobas 8000 modular analyser, Roche Diagnostics GmbH, Mannheim, Germany) was used to determine serum glucose, total cholesterol, low-density lipoprotein (LDL), high-density lipoprotein (HDL) and triacylglycerol levels. Quantification of serum 1,25-dihydroxyvitamin D [1,25(OH)2D] involved an automated chemiluminescence system (Cobas e 801 analytical module, Roche Diagnostics GmbH, Mannheim, Germany) and deficiency was defined as circulating 1,25(OH)2D < 18 pg/mL [40]. Levels of plasma ascorbic acid (vitamin C) were measured by reversed-phase high performance liquid chromatography (HPLC), as has been previously described [32]. Concentration less than 1.94 mg/L determined vitamin C deficiency [41]. Plasma malondialdehyde (MDA) levels were measured to assess lipid peroxidation based on the method of Placer et al. [42]. A red pigment was formed by the reaction of MDA with thiobarbituric acid, and the absorbance was read at 532 nm. Plasma levels of MDA were expressed as nmol/mL.

### 2.5. Primary Outcome and Sample Size Calculation

Our primary outcome was the detection of a significant increase of the Mediterranean diet adherence (assessed by the MD score) and the global health, quality of life (assessed by EORTC-QLQ C30) in the CDSS arm compared to control at study endpoint. A minimum sample size of 32 patients (16 per arm) was sufficient to result in a clinically important difference of 4 in MD [standard deviation of mean (SD) = 4] and 10 in global health, quality of life (SD = 10) using a two-tailed t test with 80% power and a 5% level of significance. Secondary outcomes were significant changes in nutrient intake (i.e., saturated fatty acids, monounsaturated fatty acids, dietary fibers and vitamin C), cardiovascular risk factors including BW, WC, BMI, total serum cholesterol, HDL cholesterol, LDL cholesterol and triglycerides acids and lipid peroxidation (i.e., MDA levels).

### 2.6. Statistical Analysis 

The SPSS statistical package (version 21.0, SPSS, Inc, ΙΒΜ, Chicago, IL, USA) was used to conduct data analysis. Descriptive statistics was performed for all assessed markers and the Kolmogorov-Smirnov test was implemented to investigate normality of distribution. Continuous variables were presented as mean values ± SD and quantitative variables as absolute and relative frequencies. For the comparison of mean changes between the control and intervention group we computed the Student’s t-test for normally distributed variables or the Mann–Whitney U test for those not normally distributed. Intra-group differences were detected by the paired samples t-test or the Wilcoxon test. Level of statistical significance was set at 0.05.

## 3. Results

**Figure 1:**
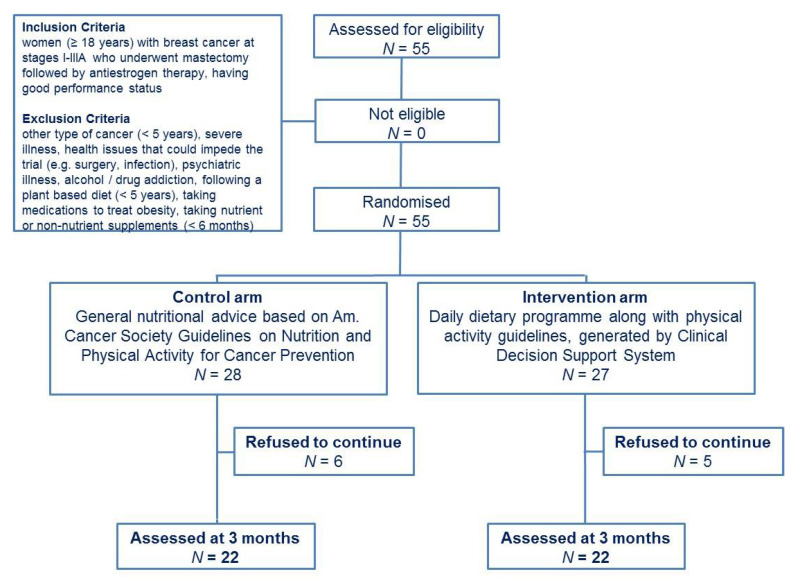
Trial workflow.

Fifty-five BC patients (*n* = 55) met our criteria for recruitment. During the trial, 11 patients (20%) refused to continue reporting unable to comply with the protocol. Finally, 22 patients in each group (Control and CDSS group) were included in final analyses (Figure 1). All participants had Greek nationality and were residents of Attica (Greece). As shown in Table 1, all volunteers reported a good performance status as indicated by “0” ECOG PS scoring. Moreover, 29.5% of enrolled participants were overweight, 39.6% were obese and 29.5% were current smokers. It is worth mentioning that 15 (68.2%) women of the control arm and 16 (72.7%) of the intervention group were postmenopausal at cancer diagnosis, but throughout the intervention all women were at a postmenopausal state. 

At baseline (0 months), no significant differences were evident between the two BC arms in regards to anthropometrics, blood markers and dietary intake (Table 1). Reported data on health-related quality of life and psychological distress are shown in Table 2.

### 3.1. Dietary Intake and Circulating Vitamin C

At follow up (3 months), the CDSS group showed greater MedDietScore, higher intake of monounsaturated fatty acids (MUFAs) and elevated fiber consumption compared to baseline (Table 3). On the other hand, the intake of saturated fatty acids (SFAs) was significantly reduced (*p* < 0.001). With regards to antioxidant vitamin C, the CDSS arm demonstrated greater dietary intake (*p* = 0.018) at 3 compared to 0 months, and this increase was significantly different from control group (*p* = 0.001).

### 3.2. Anthropometrics and Physical Activity

As indicated in Table 3, body weight, BMI, BFM% and WC of enrolled patients in the CDSS arm decreased significantly compared to control group (*p* < 0.001) at the endpoint of the trial. On the contrary, physical activity levels of the CDSS group were raised at 3 months compared to time 0 (*p* = 0.001). This change remained significant when compared to control group (*p* = 0.001), in which physical activity levels remained unaltered throughout the trial (*p* = 0.696).

### 3.3. Health Related Quality of Life and Psychological Distress

Table 4 shows changes in quality of life throughout the study period for both the control and CDSS groups. No significant differences were observed between the two arms for any assessed parameter at the trial endpoint. Nevertheless, we recorded the following statistically significant changes from the beginning to the end of the study in the CDSS group: (A) Increase in EORTC-QLQ-C30 global health-quality of life scale (*p* = 0.035). (B) Increase in EORTC-QLQ-C30 role functioning subscale (*p* = 0.047). (C) Increase in EORTC-QLQ-C30 emotional functioning subscale (*p* = 0.037). (D) Reduction in HADS depression scale (*p* = 0.022). (E) Reduction in HADS-anxiety scale (*p* = 0.022). 

### 3.4. Blood Markers

Serum glucose and triacylglycerol concentrations of the control group (Table 3) increased significantly during the intervention period (*p* = 0.046 and *p* = 0.016, respectively) but did not change in patients of the CDSS arm who followed a personalised Mediterranean dietary programme (*p* = 0.745 and *p* = 0.276, respectively). Mean changes of serum glucose and triacylglycerol concentration differed significantly between arms (*p* = 0.043 and *p* = 0.008, respectively). Blood levels of total cholesterol and LDL in the control arm were also higher at follow up compared to baseline (*p* = 0.045 and *p* = 0.037, respectively), but without reaching statistically significant differences between groups (*p* = 0.091 and *p* = 0.215, respectively). During the trial, serum HDL showed a significant rise in the CDSS group (*p* = 0.034) and remained the same in patients of the control group (*p* = 0.571). With regard to markers of oxidative stress (Table 3), a significant augmentation of MDA levels was observed at 3 months in the control compared to CDSS arm (*p* = 0.007). On the same time, concentration of plasma vitamin C was kept steady in the control group (*p* = 0.837) but was ascended in the intervention group (*p* < 0.001). Mean changes between the two arms were significantly different (*p* = 0.021).

## 4. Discussion

In the present randomised controlled study, we evaluated for the first time, the effectiveness of a clinical decision support system (CDSS) in implementing lifestyle modifications to breast cancer (BC) outpatients during the first wave of Covid-19 pandemic in Greece. According to individuals’ incorporated data, the CDSS generated a personalised nutritional plan based on the Mediterranean diet (MD) coupled with physical activity guidelines (CDSS group). Our main outcome was a significantly increased MD adherence in the CDSS arm compared to control group who received general lifestyle guidelines. At 3 months, patients of the CDSS arm achieved better health related quality life, lower body weight (nearly 5%) and body fat mass, as well as regulated fasting blood glucose and lipid levels as compared to study initiation.

The MD is considered as a modifiable lifestyle factor that could affect the development of cancer [43]. In regards to BC, the potential protective effects of MD have attracted great attention, since prevalence of BC has been lower in the Mediterranean region than in Northern or Central European countries or the US [44]. Data from observational cohort studies suggest that adherence to MD is protective against BC development [45,46,47,48]. In the PREDIMED randomised controlled trial, which included 4282 women (60–80 years) being at high risk of developing cardiovascular diseases, supplementation of MD with extra-virgin oil was beneficial in the prevention of primary BC [49]. 

Furthermore, there is evidence that MD exerts beneficial effects in women already diagnosed with BC. In a recently published observational cohort study, 1453 BC women in northern Italy were followed-up for 15 years after initial diagnosis [50]. Results showed that patients being adherent to MD had a significantly better overall survival and lower all-cause mortality compared to those with poor MD adherence. This observed association between MD adherence and low overall mortality was more profound in older (≥ 55 years of age), postmenopausal and overweight/obese women. To this end, dietary fat reduction (fats, oils, sweets) and subsequent decrease of body weight in postmenopausal women with stage I or II BC, have shown to improve relapse-free survival rate [51]. The favorable effects of MD on disease prognosis are probably attributed to the additive health benefits of its nutritional components [52]. High intake of dietary fiber has been strongly associated with a significant reduction in both all-cause and BC-specific mortality [53]. Elevated dietary intake of antioxidant vitamin C is beneficial on BC prognosis as well [54]. In our randomised controlled study, BC patients following a personalised Mediterranean dietary plan (CDSS group) had significantly higher MedDietScore compared to patients of the control group. The intervention arm showed significant greater intakes of monounsaturated fatty acids, dietary fiber and ascorbic acid than the control arm. In addition, consumption of saturated fatty acids was lower in the control arm. At the trial endpoint, the significant improvements in anthropometric characteristics of BC patients following MD (i.e. BW, BFM and WC) were accompanied by regulation of fasting blood glucose, total cholesterol, LDL and triacylglycerol levels. Physical activity levels (expressed as MET-mins/week) and blood HDL concentration of the CDSS arm were significantly elevated at the end compared to the beginning of the trial, confirming the beneficial effects of lifestyle modification in breast cancer [55]. These potential beneficial effects of MD may be explained by a reduction in oxidative damage to lipids and DNA [56]. In the present study, patients of the CDSS arm showed lower lipid peroxidation (expressed as MDA levels) and higher levels of circulating antioxidant vitamin C than the control arm.

It is well confirmed that cancer patients report declined functional capacity, increased pain and generally reduced quality of life, which may deteriorate treatment efficacy and lead to poor disease prognosis [57]. Dietary and lifestyle modification have been reported to ameliorate quality of life aspects in BC patients [57]. Likewise in our study, patients following MD for 3 months reported significant improvements in global health/quality of life, role functioning, emotional functioning, depression and anxiety, as well as trends of improvement in cognitive functioning, pain, insomnia, constipation and systemic therapy side effects. 

All in all, the use of a food database CDSS might be a promising tool for implementing a personalised dietary plan and lifestyle changes to breast cancer patients during challenging periods such as the COVID-19 pandemic minimising personal visits to healthcare settings. In this COVID era, CDSS could enable the rise of virtual consultation, always within the scope for clinical effectiveness. Nevertheless, software technology has been successfully applied in the primary care before the virus pandemic [58]. CDSS have been used to assist clinicians in nutritional screening [58], as well as in promoting healthy weight management and prevention of cardiovascular diseases [24,25,26]. Taken these together with our results, CDSS are promising tools in assisting: (a) clinicians to provide nutritional care and (b) patients to engage with lifestyle modifications.

Study limitations: We recognise that the small sample size in our study may limit the power of analysis. However, all patients were consecutively recruited implementing strict inclusion / exclusion criteria to avoid bias. Furthermore, the high dropout rate (20%) may have caused violation of the randomisation principles and consequently alterations in the effect size. To this point, an intention-to-treat-analysis would be relevant. Nevertheless, per-protocol-analysis was conducted at it is preferred in small sample sized trials to quantify actual treatment effect [59]. For these reasons, results should be interpreted with caution. The outcomes of the present work are in agreement with previous large-scale studies in breast cancer women, which point out the potential health benefits of MD. Patients with limited computer skills may face difficulties in using the CDSS. To overcome this, all patients of the intervention group were properly trained and assisted when needed. We are also aware of the emerging concerns in controlled nutritional interventions, such as treatment contamination in the control group [60]. Finally, yet importantly, the use of self-reported tools such as food diaries could be a source of bias. In our study, all self-reporting tools were already validated. Furthermore, the appointed dieticians were well experienced being able to detect unclear records and ask patients for proper clarifications.

## 5. Conclusions

We showed for the first time that the use of a food database clinical decision support system (CDSS) could be a promising tool to assist breast cancer patients to comply with dietary and lifestyle modifications during the COVID-19 pandemic. Based on individuals’ needs, the CDSS produced a nutritional plan that was in line with the Mediterranean dietary pattern, together with physical activity guidelines. Following the CDSS programme for 3 months, significant ameliorations in Mediterranean diet adherence, quality of life, as well as anthropometrics and blood indices were recorded. During challenging periods such as the COVID-19 pandemic, the application of CDSS could promote lifestyle changes in breast cancer patients minimising personal visits to healthcare settings and the risk to infection. To this end, larger-scale population studies are needed to investigate the potential beneficial effects of software-based technologies on the nutritional care of vulnerable populations such as cancer patients.

## Figures and Tables

**Table 1:** Baseline characteristics of recruited BC patients.

Characteristics	Enrolled Patients (*N* = 44)	Control Group (*N* = 22)	CDSS Group (*N* = 22)	*p*
Females	44	22	22	-
Age (years)	49.7 ± 8.1	49.8 ± 8.4	49.6 ± 7.9	0.956
Body weight (Kg)	75.8 ± 13.9	73.2 ± 13.1	78.4 ± 14.6	0.218
BMI (kg/m^2^) <18.5 18.5–24.9 25–29.9 >30	28.8 ± 5.6 0 14 (31.8) 13 (29.5) 17 (38.6)	28.3 ± 6.0 0 9 (40.9) 6 (27.3) 7 (31.8)	29.3 ± 5.4 0 5 (22.7) 7 (31.8) 10 (45.4)	0.556
% BFM	39.4 ± 8.2	38.3 ± 8.4	40.6 ± 8.0	0.326
WC (cm)	97.9 ± 11.7	96.9 ± 10.6	98.8 ± 12.9	0.593
Hormone Therapy Aromatase inhibitors Tamoxifen	30 (68.2) 14 (31.8)	16 (72.7) 6 (27.3)	14 (63.6) 8 (36.4)	-
Current smokers	13 (29.5)	6 (27.3)	7 (31.8)	-
ECOG performance status Score 0 Score 1	44 (100.0) 0	22 (100.0) 0	22 (100.0) 0	-
METs-min/week	767.9 ± 410.7	841.5 ± 445.9	694.4 ± 367.8	0.239
Glucose (mg/dL)	94.8 ± 10.1	95.5 ± 10.3	94.1 ± 10.1	0.655
Total cholesterol (mg/dL)	192.3 ± 40.7	189.1 ± 47.5	195.4 ± 33.4	0.611
HDL (mg/dL)	58.2 ± 19.1	58.4 ± 17.1	58.0 ± 21.3	0.942
LDL (mg/dL)	114.8 ± 31.8	115.6 ± 35.9	113.9 ± 27.8	0.856
Triacylglycerols (mg/dL)	94.5 ± 38.9	93.4 ± 36.3	95.6 ± 42.1	0.850
MDA (nmol/mL)	1.5 ± 0.5	1.5 ± 0.5	1.6 ± 0.5	0.669
Vitamin C (mg/L)	4.4 ± 2.1	4.1 ± 2.2	4.7 ± 2.1	0.285
Vitamin 1,25(OH)_2_D (ng/L)	31.9 ± 6.8	32.9 ± 8.9	31.0 ± 3.6	0.363
MedDietScore	31.0 ± 4.0	31.3 ± 3.5	30.6 ± 4.5	0.552
Fibers (g/day)	18.7 ± 2.6	18.7 ± 2.8	18.7± 2.6	0.987
SFAs (g/day)	16.8 ± 3.2	17.3 ± 3.7	16.3 ± 2.7	0.312
MUFAs (g/day)	29.8 ± 4.7	30.1 ± 5.2	29.5 ± 4.4	0.689
Vitamin C (mg/day)	267.2 ± 81.6	281.8 ± 99.5	252.7 ± 57.4	0.242

Data are presented as counts (percentages) or means ± standard deviation (SD). *p*: significant differences between the Control and the CDSS arm resulted from the independent sample *t*-test or, where appropriate, the Mann–Whitney test. Level of statistical significance was set at 0.05. BC, breast cancer; CDSS, clinical decision support system; BMI, body mass index; BFM, body fat mass; WC, waist circumference; ECOG, Eastern Cooperative Oncology Group; METs, metabolic equivalents; HDL, high density lipoprotein; LDL, low density lipoprotein; MDA, malondialdehyde; SFAs, saturated fatty acids; MUFAs, monounsaturated fatty acids.

**Table 2:** Health-related quality of life, anxiety and depression scales at baseline.

Scales	Control Group (*N* = 22)	CDSS Group (*N* = 22)	*p*
EORTC-QLQ-C30: functional scales			
Physical	75.8 ± 20.3	73.9 ± 13.9	0.732
Role	56.8 ± 31.6	53.0 ± 35.5	0.710
Emotional	64.0 ± 22.8	64.4 ± 26.6	0.959
Cognitive	74.2 ± 18.3	72.0 ± 31.4	0.770
Social	78.8 ± 29.6	75.8 ± 17.6	0.683
EORTC-QLQ-C30: symptom scales			
Fatigue	46.5 ± 20.2	48.5 ± 28.4	0.787
Nausea / vomiting	1.5 ± 7.1	1.5 ± 4.9	0.998
Pain	31.1 ± 31.4	31.8 ± 25.7	0.931
Dyspnoea	28.8 ± 25.8	24.2 ± 27.6	0.576
Insomnia	34.8 ± 34.9	33.3 ± 38.5	0.892
Appetite loss	10.6 ± 15.9	7.6 ± 14.3	0.510
Constipation	12.1 ± 28.3	13.6 ± 24.5	0.850
Diarrhoea	12.1 ± 26.3	12.1 ± 21.9	0.999
EORTC-QLQ-C30: global health, QoL	61.0 ± 22.6	62.9 ± 18.5	0.762
EORTC-QLQ-BR23: functional scales			
Body image	67.0 ± 26.9	69.7 ± 27.6	0.748
Sexual functioning	79.5 ± 24.1	79.5 ± 26.2	0.999
Future perspective	36.4 ± 32.4	40.9 ± 32.4	0.644
EORTC-QLQ-BR23: symptoms			
Systemic therapy side effects	14.5 ± 9.8	15.8 ± 13.3	0.715
Breast symptoms	28.0 ± 26.8	31.1 ± 26.4	0.707
Arm symptoms	26.8 ± 23.2	28.8 ± 26.7	0.789
HADS: depression 0 to 7 (%) 8 to 10 (%) 11 to 21 (%)	6.5 ± 4.0 13 (59.1) 6 (27.3) 3 (13.6)	6.6 ± 3.1 11 (50.0) 10 (45.5) 1 (4.5)	0.866
HADS: anxiety 0 to 7 (%) 8 to 10 (%) 11 to 21 (%)	9.4 ± 5.1 10 (45.5) 3 (13.6) 9 (40.9)	8.5 ± 4.9 10 (45.5) 4 (18.2) 8 (36.4)	0.567

Data are presented as counts (percentages) or means ± standard deviation (SD). *p*: significant differences between the Control and the CDSS arm resulted from the independent sample *t*-test or, where appropriate, the Mann–Whitney test. Level of statistical significance was set at 0.05. BC, breast cancer; CDSS, clinical decision support system; EORTC-QLQ-C30 or BR-23, European Organization for Research and Treatment of Cancer Quality of Life Questionnaire Core-30 or Breast-23 respectively; HADS, Hospital Anxiety and Depression Scale; QoL, quality of life.

**Table 3:** Characteristics of BC patients at baseline and study end (3 months).

Characteristics.	Group	Baseline (*N* = 22)	3 Months (*N* = 22)	*p*	* *p*
		Mean ± SD	Mean ± SD		
Body weight (kg)	control	73.2 ± 13.1	74.1 ± 13.5	0.223	**<0.001**
	CDSS	78.4 ± 14.6	74.8 ± 13.4	**<0.001**	
BMI (kg/m^2^) <18.5 18.5–24.99 25–30 >30	control	28.3 ± 6.00 9 (40.9) 6 (27.3) 7 (31.8)	28.6 ± 6.00 6 (27.3) 10 (45.4) 6 (27.3)	0.262	**<0.001**
	CDSS	29.3 ± 5.40 5 (22.7) 7 (31.8) 10 (45.4)	28.0 ± 4.90 7 (31.8) 8 (36.4) 7 (31.8)	**<0.001**	
% BFM	control	38.3 ± 8.4	39.0 ± 8.1	0.183	**<0.001**
	CDSS	40.6 ± 8.0	37.2 ± 8.0	**<0.001**	
WC (cm)	control	96.9 ± 10.6	97.3 ± 10.8	0.579	**<0.001**
	CDSS	98.8 ± 12.9	95.7 ± 12.2	**<0.001**	
METs-min/week	control	841.5 ± 445.9	798.2 ± 668.8	0.696	**0.001**
	CDSS	694.4 ± 367.8	1393.4 ± 895.9	**0.001**	
Current smokers	control	6 (27.3)	4 (18.2)	-	-
	CDSS	7 (31.8)	4 (18.2)	-	
Glucose (mg/dL)	control	95.5 ± 10.3	104.6 ± 23.5	**0.046**	**0.043**
	CDSS	94.1 ± 10.1	93.6 ± 7.0	0.745	
Cholesterol (mg/dL)	control	189.1 ± 47.5	205.3 ± 48.9	**0.045**	0.091
	CDSS	195.4 ± 33.4	193.1 ± 35.8	0.758	
HDL (mg/dL)	control	58.4 ± 17.1	60.2 ± 15.1	0.571	0.114
	CDSS	58.0 ± 21.3	69.1 ± 20.7	**0.034**	
LDL (mg/dL)	control	115.6 ± 35.9	127.6 ± 37.7	**0.037**	0.215
	CDSS	113.9 ± 27.8	114.5 ± 39.4	0.936	
Triacylglycerols (mg/dL)	control	93.4 ± 36.3	112.8 ± 49.0	**0.016**	**0.008**
	CDSS	95.6 ± 42.1	89.8 ± 36.3	0.276	
MDA (nmol/mL)	control	1.5 ± 0.5	1.8 ± 0.6	**0.017**	**0.007**
	CDSS	1.6 ± 0.5	1.3 ± 0.5	0.144	
Vitamin C (mg/L)	control	4.1 ± 2.2	4.2 ± 1.4	0.837	**0.021**
	CDSS	4.7 ± 2.1	6.1 ± 1.9	**<0.001**	
Vitamin 1,25(OH)_2_D (ng/L)	control	32.9 ± 8.9	30.9 ± 8.8	0.163	0.066
	CDSS	31.0 ± 3.6	33.0 ± 7.5	0.224	
MedDietScore	control	31.3 ± 3.5	31.9 ± 4.0	0.409	**0.002**
	CDSS	30.6 ± 4.5	34.6 ± 4.3	**<0.001**	
Fibers (g/day)	control	18.7 ± 2.8	19.1 ± 3.1	0.089	**0.003**
	CDSS	18.7 ±2.6	20.8 ± 3.5	**<0.001**	
SFAs (g/day)	control	17.3 ± 3.7	18.1 ± 3.8	**<0.001**	**0.001**
	CDSS	16.3 ± 2.7	14.6 ± 2.5	**0.017**	
MUFAs (g/day)	control	30.1 ± 5.2	29.6 ± 5.5	0.628	**<0.001**
	CDSS	29.5 ± 4.4	33.3 ± 3.1	**<0.001**	
Vitamin C (mg/day)	control	281.8 ± 99.5	236.8 ± 67.6	**0.005**	**0.001**
	CDSS	252.7 ± 57.4	298.2 ± 73.6	**0.018**	

Data are presented as counts (percentages) or means ± standard deviation (SD). *p*: significant intra-group differences resulted from the paired samples *t* test or, where appropriate, the Wilcoxon test. * *p*: significant changes between groups at follow up resulted from the independent samples *t*-test or, where appropriate, the Mann–Whitney test. Level of statistical significance was set at 0.05. Significant *p* are bold. BC, breast cancer; CDSS, clinical decision support system; BMI, body mass index; BFM, body fat mass; WC, waist circumference; METs, metabolic equivalents; HDL, high density lipoprotein; LDL, low density lipoprotein; MDA, malondialdehyde; SFAs, saturated fatty acids; MUFAs, monounsaturated fatty acids.

**Table 4:** Health-related quality of life, anxiety and depression scales at baseline and study end (3 months).

Scales	Group	Baseline (*N* = 22)	3 Months (*N* = 22)	*p*	* *p*
		Mean ± SD	Mean ± SD		
EORTC-QLQ-C30: functional scales					
Physical	control	75.8 ± 20.3	74.8 ± 19.3	0.836	0.361
	CDSS	73.9 ± 13.9	77.9 ± 11.5	0.200	
Role	control	56.8 ± 31.6	56.8 ± 28.0	0.999	0.186
	CDSS	53.0 ± 35.5	68.9 ± 21.4	**0.047**	
Emotional	control	64.0 ± 22.8	70.1 ± 18.5	0.292	0.542
	CDSS	64.4 ± 26.6	75.0 ± 12.9	**0.037**	
Cognitive	control	74.2 ± 18.3	75.8 ± 21.7	0.808	0.212
	CDSS	72.0 ± 31.4	84.8 ± 11.4	0.060	
Social	control	78.8 ± 29.6	78.8 ± 21.3	0.999	0.830
	CDSS	75.8 ± 17.6	77.3 ± 15.0	0.677	
EORTC-QLQ-C30: symptoms					
Fatigue	control	46.5 ± 20.2	41.4 ± 28.1	0.370	0.662
	CDSS	48.5 ± 28.4	39.9 ± 24.6	0.157	
Nausea / vomiting	control	1.5 ± 7.1	2.3 ± 7.8	0.747	0.999
	CDSS	1.5 ± 4.9	2.3 ± 5.9	0.329	
Pain	control	31.1 ± 31.4	24.2 ± 32.0	0.387	0.802
	CDSS	31.8 ± 25.7	22.7 ± 28.0	0.062	
Dyspnoea	control	28.8 ± 25.8	22.7 ± 23.9	0.296	0.609
	CDSS	24.2 ± 27.6	22.7 ± 28.0	0.825	
Insomnia	control	34.8 ± 34.9	30.3 ± 27.0	0.601	0.357
	CDSS	33.3 ± 38.5	18.2 ± 24.6	0.057	
Appetite loss	control	10.6 ± 15.9	9.1 ± 15.2	0.329	0.481
	CDSS	7.6 ± 14.3	3.0 ± 9.8	0.266	
Constipation	control	12.1 ± 28.3	10.6 ± 21.5	0.747	0.216
	CDSS	13.6 ± 24.5	3.0 ± 9.8	0.069	
Diarrhoea	control	12.1 ± 26.3	6.1 ± 22.1	0.406	0.870
	CDSS	12.1 ± 21.9	4.5 ± 11.7	0.203	
EORTC-QLQ-C30: Global health, QoL	control	61.0 ± 22.6	67.0 ± 16.6	0.179	0.613
	CDSS	62.9 ± 18.5	72.0 ± 11.9	**0.035**	
EORTC-QLQ-BR23: functional scales					
Body image	control	67.0 ± 26.9	67.8 ± 23.6	0.891	0.745
	CDSS	69.7 ± 27.6	68.2 ± 23.1	0.725	
Sexual functioning	control	79.5 ± 24.1	70.5 ± 29.1	0.063	0.127
	CDSS	79.5 ± 26.2	81.1 ± 23.2	0.765	
Future perspective	control	36.4 ± 32.4	36.4 ± 27.0	0.999	0.137
	CDSS	40.9 ± 32.4	56.1 ± 21.5	0.116	
EORTC-QLQ-BR23: symptoms					
Systemic therapy side effects	control	14.5 ± 9.8	13.9 ± 13.2	0.846	0.900
	CDSS	15.8 ± 13.3	14.7 ± 12.9	0.056	
Breast symptoms	control	28.0 ± 26.8	25.4 ± 22.6	0.050	0.901
	CDSS	31.1 ± 26.4	27.7 ± 20.1	0.568	
Arm symptoms	control	26.8 ± 23.2	28.8 ± 18.4	0.296	0.395
	CDSS	28.8 ± 26.7	26.3 ± 22.1	0.613	
HADS: depression 0 to 7 (%) 8 to 10 (%) 11 to 21 (%)	control	6.5 ± 4.0 13 (59.1) 6 (27.3) 3 (13.6)	5.5 ± 3.7 19 (86.4) 2 (9.1) 1 (4.5)	0.338	0.330
	CDSS	6.6 ± 3.1 11 (50.0) 10 (45.5) 1 (4.5)	4.4 ± 3.8 16 (72.7) 4 (18.2) 2 (9.1)	**0.022**	
HADS: anxiety 0 to 7 (%) 8 to 10 (%) 11 to 21 (%)	control	9.4 ± 5.1 10 (45.5) 3 (13.6) 9 (40.9)	7.1 ± 5.5 12 (54.5) 4 (18.2) 6 (27.3)	0.089	0.848
	CDSS	8.5 ± 4.9 10 (45.5) 4 (18.2) 8 (36.4)	6.0 ± 3.6 15 (68.2) 4 (18.2) 3 (13.6)	**0.022**	

Data are presented as counts (percentages) or means ± standard deviation (SD). *p*: significant intra-group differences resulted from the paired samples t test or, where appropriate, the Wilcoxon test. * *p*: significant changes between groups at follow up resulted from the independent samples t-test or, where appropriate, the Mann–Whitney test. Level of statistical significance was set at 0.05. Significant *p* are bold. BC, breast cancer; CDSS, clinical decision support system; EORTC-QLQ-C30 or BR-23, European Organization for Research and Treatment of Cancer Quality of Life Questionnaire Core-30 or Breast-23 respectively; HADS, Hospital Anxiety and Depression Scale; QoL, quality of life.

## Supplementary Materials

The following are available online at https://www.mdpi.com/article/10.3390/nu13062115/s1, Figure S1: Energy requirements assessed by the clinical decision support system (CDSS), Figure S2: Physical activity status assessed by the clinical decision support system (CDSS), Figure S3: Nutritional status assessed by the clinical decision support system (CDSS).
