# Supplementary material for: Application of Clinical Decision Support System to Assist Breast Cancer Patients with Lifestyle Modifications during the COVID-19 Pandemic: A Randomised Controlled Trial
Source: Nutrients. 2021 Jun 20;13(6):2115. doi: 10.3390/nu13062115 (PMC8235424; doi:10.3390/nu13062115)
Supplement: Supplementary file 1 [file nutrients-13-02115-s001.zip › nutrients-1241044-supplementary.pdf]

Supplementary Material

Application of Clinical Decision Support System to assist breast cancer patients with lifestyle modifications during the COVID-19 pandemic: a randomised controlled trial

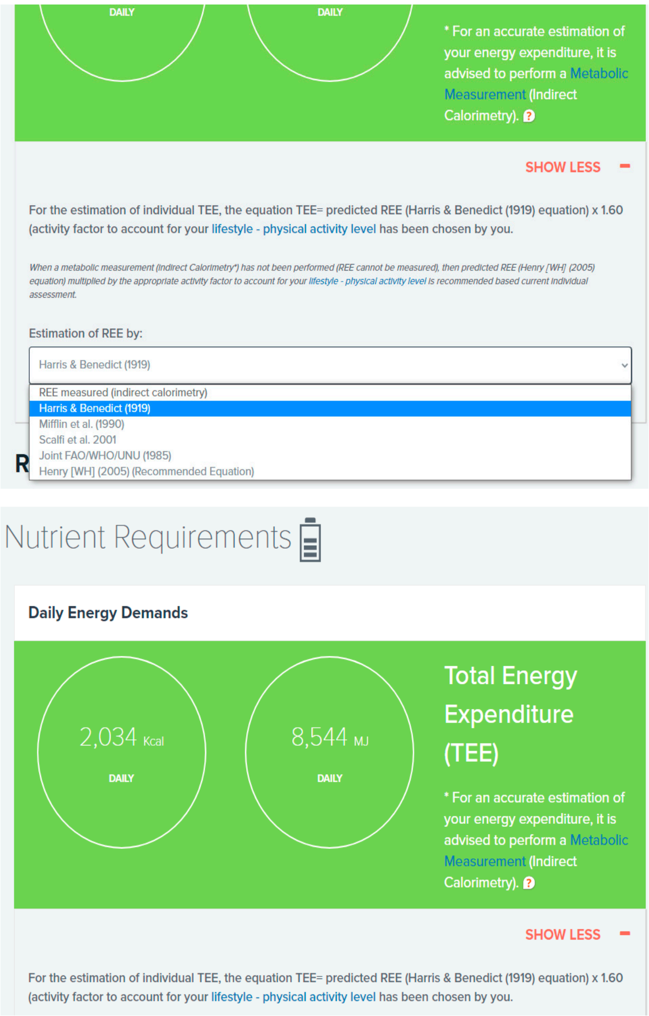

Figure S1. Energy requirements assessed by the clinical decision support system (CDSS).

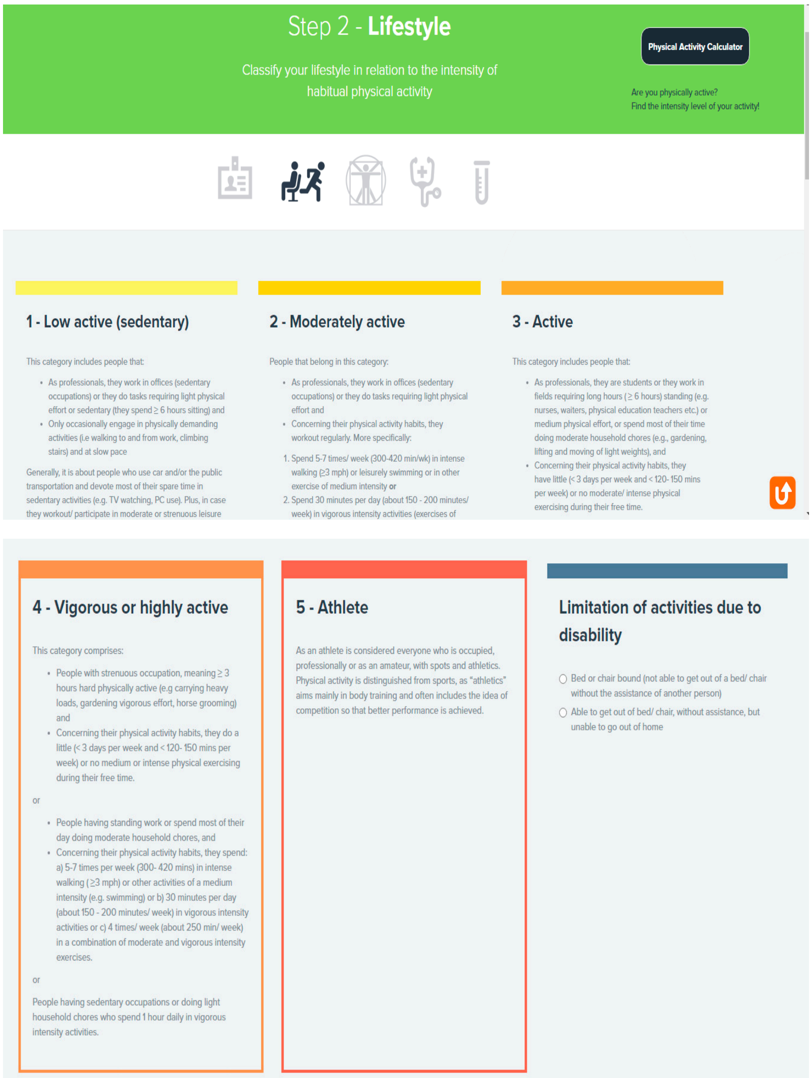

Figure S2. Physical activity status assessed by the clinical decision support system (CDSS).

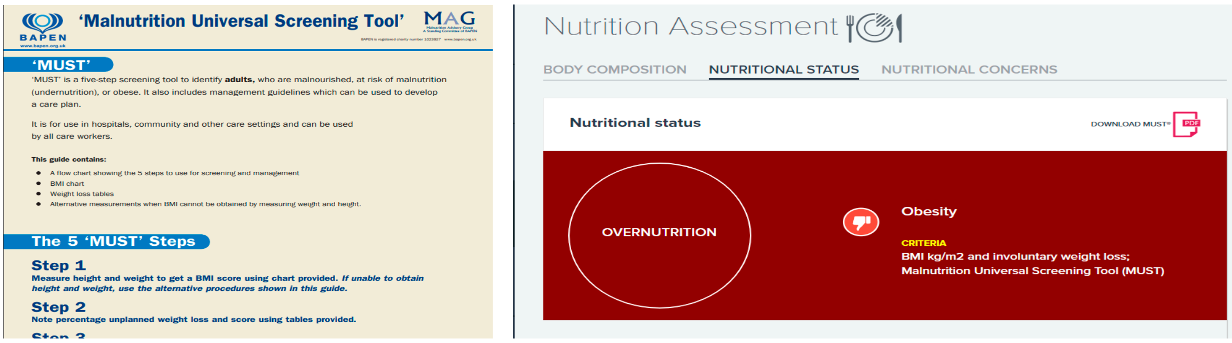

Figure S3. Nutritional status assessed by the clinical decision support system (CDSS) .
